# Homeostatic Control of the Thyroid–Pituitary Axis: Perspectives for Diagnosis and Treatment

**DOI:** 10.3389/fendo.2015.00177

**Published:** 2015-11-20

**Authors:** Rudolf Hoermann, John E. M. Midgley, Rolf Larisch, Johannes W. Dietrich

**Affiliations:** ^1^Department of Nuclear Medicine, Klinikum Luedenscheid, Luedenscheid, Germany; ^2^North Lakes Clinical, Ilkley, UK; ^3^Medical Department I, Endocrinology and Diabetology, Bergmannsheil University Hospitals, Ruhr University of Bochum, Bochum, Germany; ^4^Ruhr Center for Rare Diseases (CeSER), Ruhr University of Bochum and Witten/Herdecke University, Bochum, Germany

**Keywords:** homeostasis, feedback regulation, TSH, thyroid hormones, deiodinase, set point

## Abstract

The long-held concept of a proportional negative feedback control between the thyroid and pituitary glands requires reconsideration in the light of more recent studies. Homeostatic equilibria depend on dynamic inter-relationships between thyroid hormones and pituitary thyrotropin (TSH). They display a high degree of individuality, thyroid-state-related hierarchy, and adaptive conditionality. Molecular mechanisms involve multiple feedback loops on several levels of organization, different time scales, and varying conditions of their optimum operation, including a proposed feedforward motif. This supports the concept of a dampened response and multistep regulation, making the interactions between TSH, FT4, and FT3 situational and mathematically more complex. As a homeostatically integrated parameter, TSH becomes neither normatively fixed nor a precise marker of euthyroidism. This is exemplified by the therapeutic situation with l-thyroxine (l-T4) where TSH levels defined for optimum health may not apply equivalently during treatment. In particular, an FT3–FT4 dissociation, discernible FT3–TSH disjoint, and conversion inefficiency have been recognized in l-T4-treated athyreotic patients. In addition to regulating T4 production, TSH appears to play an essential role in maintaining T3 homeostasis by directly controlling deiodinase activity. While still allowing for tissue-specific variation, this questions the currently assumed independence of the local T3 supply. Rather it integrates peripheral and central elements into an overarching control system. On l-T4 treatment, altered equilibria have been shown to give rise to lower circulating FT3 concentrations in the presence of normal serum TSH. While data on T3 in tissues are largely lacking in humans, rodent models suggest that the disequilibria may reflect widespread T3 deficiencies at the tissue level in various organs. As a consequence, the use of TSH, valuable though it is in many situations, should be scaled back to a supporting role that is more representative of its conditional interplay with peripheral thyroid hormones. This reopens the debate on the measurement of free thyroid hormones and encourages the identification of suitable biomarkers. Homeostatic principles conjoin all thyroid parameters into an adaptive context, demanding a more flexible interpretation in the accurate diagnosis and treatment of thyroid dysfunction.

## Dual Role of Hormones in Thyroid Homeostasis

**Figure 1:**
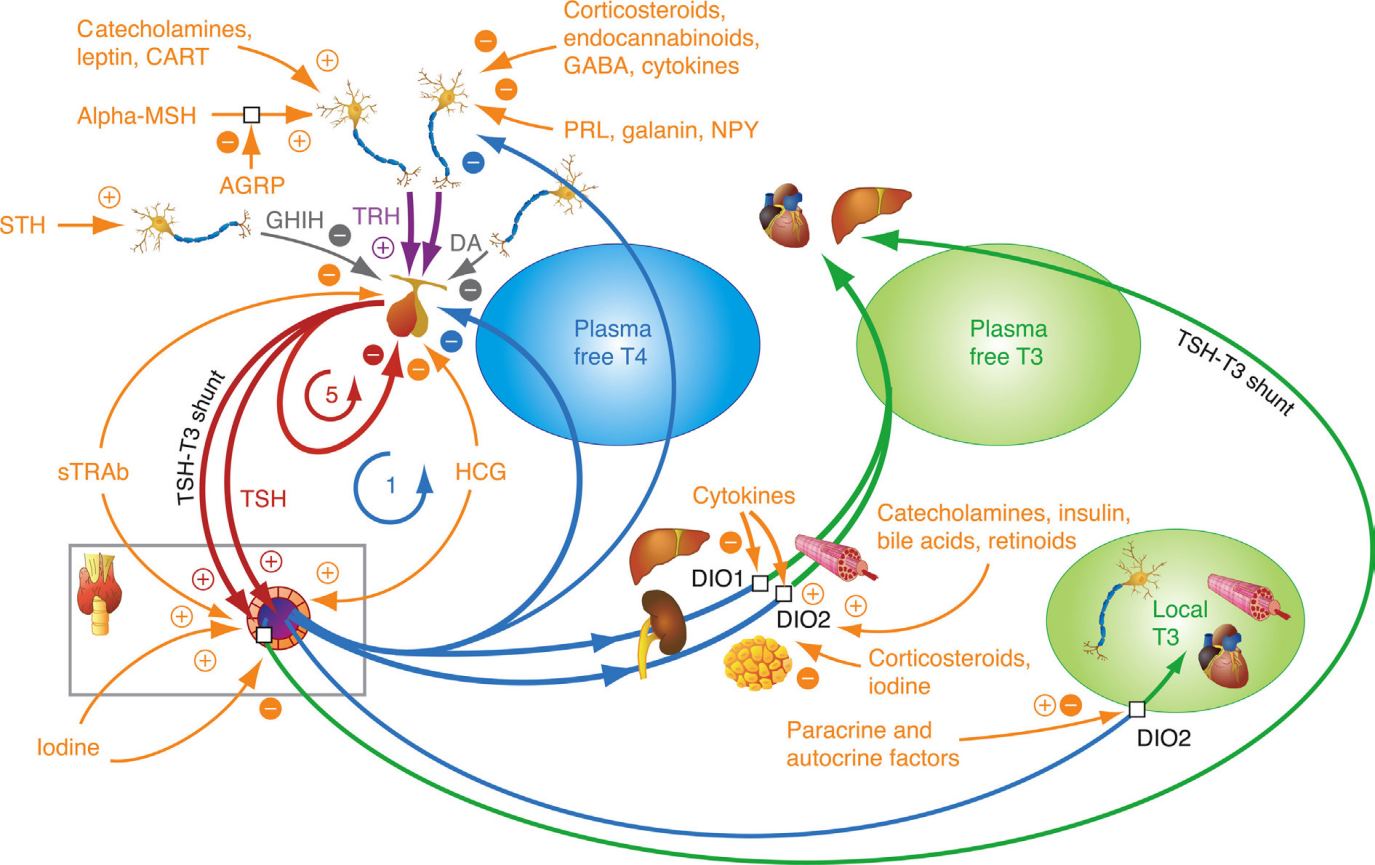
**Homeostatic integration of central, thyroidal, and peripheral influences**. The integrated control involves several major control loops, a negative feedback control of thyroid hormones on pituitary TSH and hypothalamic TRH, positive stimulatory control of TRH on TSH, ultrashort feedback of TSH on its own secretion, and feedforward control of deiodinases by TSH. Other thyrotropic agonists than TSH, such as TSH receptor antibodies (TSH-R Ab) and human chorionic gonadotropin (hCG), play an important role in diseases, such as Graves’ disease and pregnancy-related hyperthyroidism. A plethora of additional influences may fine-tune the responses at each level of organization. 1 refers to the classical Astwood–Hoskins loop, and 5 indicates ultrashort feedback loop of TSH on its own secretion, described in the text. Additional feedback loops (not shown here) control the binding of thyroid hormones to plasma proteins (8, 10).

The dynamic ability to maintain flexible homeostatic equilibria in response to environmental challenges is a hallmark of a healthy state of the organism. Thyroid hormones assume a dual role in homeostatic regulation, acting as controlling as well as controlled elements. They target a broad spectrum of metabolic effects but concomitantly are strongly regulated themselves. A basic understanding of thyroid control involving pituitary thyrotropin (TSH) has been readily exploited for the diagnosis of thyroid disorders (1–4). As a result, measurement of TSH, though an indirect indicator of thyroid homeostasis, has become central to contemporary thyroid function testing (4, 5). Our knowledge of the mechanisms involved in the regulation of thyroid hormones has greatly evolved in recent years. The underlying system is far more complex than previously thought (Figure 1). This requires a revision of long-held simplistic concepts and promotes a multifactorial concept of the feedback control between the thyroid and the pituitary gland (6–9). In this article, we review the role of thyroid homeostasis in the light of recent developments and discuss the resulting new perspectives for diagnosis and treatment of thyroid dysfunction.

## Homeostatic Aspects of Thyroid Function Control

From first principles, it is clinically important to understand clearly what distinguishes a controlling parameter from any other. A change in TSH concentration could be either merely adaptive to restore true euthyroidism or a failed attempt to maintain the euthyroid state. Corrective moves of the control parameter may therefore merely imply a change in the mechanism targeted. This depends on whether the correction sought for is successfully achieved or not. Any meaningful interpretation must respect those particularities in TSH, which do not apply to most other laboratory parameters.

The concept of a control loop feeding back information about the state of thyroid production to the pituitary gland was postulated as early as 1940 (11) and established experimentally before 1950 (12, 13). Models initially assumed an inverse linear correlation between TSH and T4 (14–17), but following more detailed analysis this was later changed to a log-linear relationship, which has remained the standard model ever since (18–22). As circulating thyroid hormones are bound to a large extent to transport proteins (TBG, transthyretin, and albumin) TSH has mostly been related to the unbound biologically active hormone, free T4 (FT4). Table 1 summarizes various thyroid–pituitary feedback models that have been proposed in the literature over the last decades (6–10, 14–19, 21–39). The feedforward path linking TSH levels to T4 output has been modeled as a simple linear relation in the majority of these models.

**Table 1:** **Historical perspective on evolving models for the TSH-T4/FT4 relationship**.

Author	Year of publication	Regression
Danziger and Elmergreen (14)	1956	Linear
Roston (23)	1959	Linear with basal secretion
Norwich and Reiter (15)	1965	Linear
DiStefano and Stear (36)	1968	Linear with basal secretion
DiStefano and Chang (16, 37)	1969 and 1971	Linear with basal secretion
Saratchandran et al. (24)	1976	Log-linear
Wilkin et al. (17)	1977	Restricted maximum secretion
Hatakeyama and Yagi (38)	1985	Power law and linear
Cohen (25)	1990	Exponential
Spencer et al. (19)	1990	Log-linear
Li et al. (26)	1995	Non-linear polynomial
Dietrich et al. (10, 27, 28, 40)	1997, 2002, and 2004	Michaelis–Menten kinetics, non-competitive inhibition, and first-order time constants
Sorribas and González (39)	1999	Power laws
Leow (18)	2007	Log-linear
Degon et al. (29)	2008	Non-linear
McLanahan et al. (30)	2008	Michaelis–Menten kinetics, non-competitive inhibition, and first-order time constants
Eisenberg et al. (31, 32)	2008 and 2010	Adopted from DiStefano
Benhadi et al. (21)	2010	Log-linear
Hoermann et al. (6, 9)	2010 and 2014	Erf (modulated log-linear) and polynomial
van Deventer et al. (22)	2011	Log-linear
Clark et al. (33)	2012	Polynomial
Midgley et al. (8)	2013	Segmented log-linear
Hadlow et al. (7)	2013	Polynomial
Jonklaas et al. (34)	2014	Segmented
Goede et al. (35, 41)	2014	Exponential (log-linear) and log-linear with Michael–Menten-type feedforward path

*Models of DiStefano et al. and Eisenberg are based on the same platform. Likewise models of Dietrich et al., McLanahan et al., Midgley et al., and Hoermann et al. (9) are based on a common formalism. The model of Goede et al. (41) inherits from those of Leow (18) and Dietrich et al. (10, 27, 28, 40)*.

From the perspective of a sufficiently sensitive defensive response, however, linear or log-linear proportional relations between TSH and FT4 would not intuitively appear to be the most adequate solution. As in many technical systems, a dampened response could be more suited to maintain the controlled parameter at a given stable level with a minimal fluctuation. This consideration requires an examination of the system operating beyond the standard log-linear model.

## A Reassessment of Thyroid–Pituitary Feedback Control

**Figure 2:**
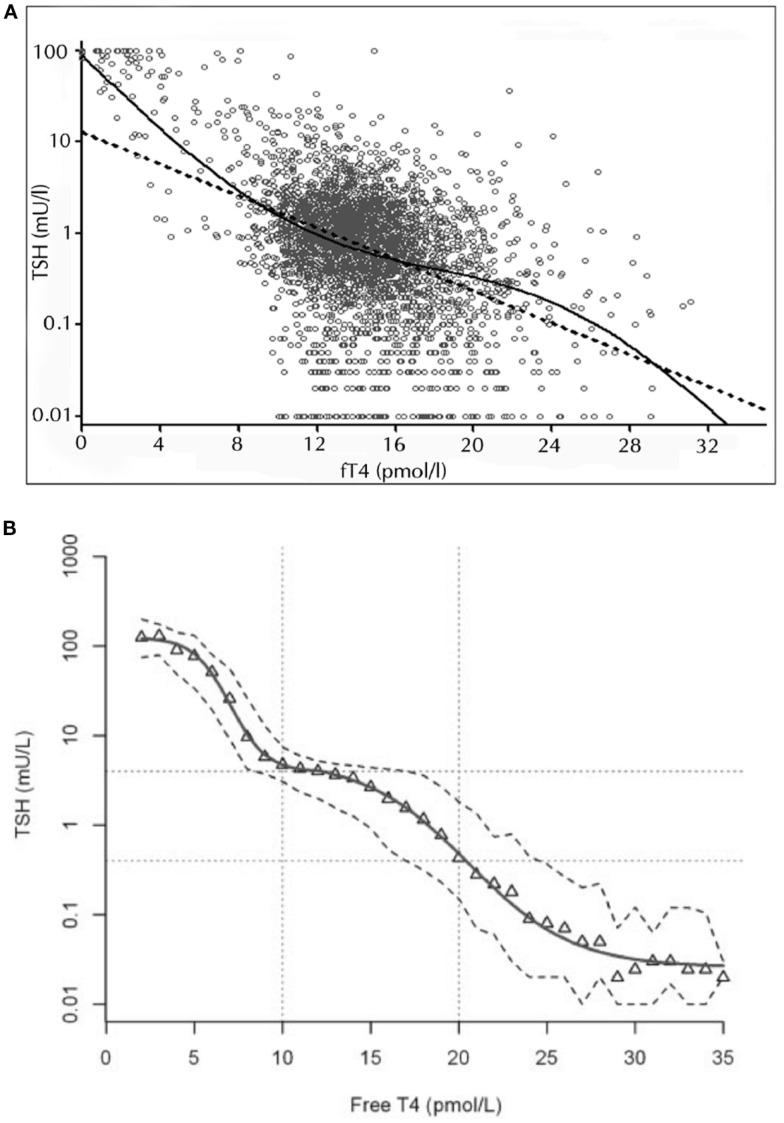
**Non-linear relationship between logTSH and free T4**. The two studies by Hoermann et al. (6) **(A)** and Hadlow et al. (7) **(B)** show that the TSH–FT4 relationship may not follow a proportional log-linear model (dashed straight line), displaying a damped response in the euthyroid range and steeper gradients at the hypothyroid or hyperthyroid spectrum. The superiority of the non-linear modulatory logistic function shown over the standard log-linear model was statistically established by a strict curve-fitting template based on Akaike’s information criterion (6). A multistep regulation of the FT4–TSH feedback control is discussed in the text. Adapted and reproduced with permission from Hoermann et al. (6) and Hadlow et al. (7).

It would be ideal to follow individuals’ responses during progression from the hypothyroid to the hyperthyroid state to study the changing pituitary response over the entire functional spectrum. Analyses that do not cover the full spectrum from the hypothyroid to the hyperthyroid extremes are problematic to interpret, because wide variations in the slopes of the logTSH–FT4 relationships have been reported (19, 21, 22, 42). Particularly, different weightings of the extreme, statistically most influential dysfunctional examples in the various patient panels impact heavily on the linear regression. Studies restricted to a narrower euthyroid panel have yielded TSH estimates when extrapolating the regression line to the hypothyroid state are much lower than those clinically observed in the hypothyroid patient (21, 22). Large cross-sectional studies have examined the TSH–FT4 relationship over the entire functional range but did not confirm a proportional and log-linear TSH–FT4 relationship, rather suggesting that the TSH response to changes in FT4 is curvilinear and damped in the middle part (6, 7, 9, 33) (Figure 2). Technically, using either a modulatory logistic function, a segmented approach, non-competitive inhibition, or polynomial approximation offers similar ways of examining the same underlying principles of a non-proportional adaptive response dependent on the actual thyroid hormone status (6–8, 33). The non-linearity of the logTSH–FT4 relationship has been independently confirmed by several groups and was replicated in a prospective study involving 1912 subjects (7–9, 33, 34). Thus, the TSH–FT4 relationship is not invariant but is impacted on by the thyroid status itself, which acts as a major determinant of the gradient relating TSH and FT4 (8). Accordingly, the thyroid state may be more vigorously defended, the greater is the deviation from a putative optimum state (6) (Figure 2). This behavior provides a far more flexible response than a simple log-linear template. It may conceivably arise from the integrated action of the multiple feedback loops operating at various levels of organization, as shown in Figure 1. The consequences of the non-proportional relationship for the clinical interpretation are discussed below.

It should be noted that these studies relied on immunometric FT4 assays, since tandem mass spectrometry (LC–MS/MS) is currently not practicable with large patient panels. However, none of the studies gave indication of a TSH–thyroid hormone mismatch. The inverse linear relation between thyroid hormones and logTSH was similarly broken with an immunoassay and LC–MS/MS in a clinically diverse sample contradicting an earlier report (22, 34). A subanalysis was conducted in a cohort of otherwise “healthy” out-patients without relevant comorbidity to ascertain FT3 or FT4 measurements and their relationships were not compromised by problematic conditions, such as pituitary dysfunction and the non-thyroid illness syndrome (6). Reliability of immunological methods and harmonization among various commercially available assays has been questioned (43, 44). In this respect, we have extensively evaluated the analytical performance of the immunoassays used in our inter-relational studies (45). In particular, we verified the reference range in the local population, demonstrated robustness of the relationships despite biological variation, and quantified other influences on the defining relationships, such as age and body mass index (9, 45). A clinically important and specific role of FT3 measurement was further supported by our pilot study in a large unselected predominantly euthyroid sample (46). FT3, in the range from 1 to 10 pmol/l, but not FT4 or TSH, showed a significant u-shaped relationship with the Hospital Anxiety and Depression Score (HADS) as well as the anxiety and depression subscales in a generalized linear-quadratic model (not a widely used, but unsuitable linear model) (46). Together these findings support suitability of FT3 for correlative studies when measured with the same instrument at a single institution but do not negate issues of insufficient validation and standardization with the methods discussed below.

## Molecular Mechanisms Involved in the Feedback Control

While this article focuses on homeostatic regulation and an in-depth review of the growing body of molecular details is beyond its scope, it should be briefly shown that key mechanisms are reconcilable with a non-proportional model. Both T3 and T4 following its conversion into T3 bind to specific intracellular TR receptors exerting a repressive action on various genes, including TSHβ and, to a lesser degree, α-subunit (47–52). Among the isoforms of TR expressed in various tissues, TRβ2 is active in the central nervous system, hypothalamus, and pituitary gland, with a reported sensitivity enhanced up to 10-fold to thyroid hormones, compared with TRβ1 (53, 54). Such a differential response should enable the central tissues to anticipate T3/T4 oversupply before it can affect less sensitive peripheral tissues. Similarly, deiodinases in central and peripheral tissues are also regulated differently, enhancing T3 conversion and providing a differentiated mechanism for oversensitively responding to changes in FT4 in the feedback loop (55–63). Specifically, type 2 deiodinase ubiquitination has recently been shown to be instrumental in hypothalamic negative feedback regulation and is expressed in a non-uniform way among various tissues (64). Contrary to earlier assumptions, T3 and T4 do not diffuse freely across the plasma membrane but are actively transported by specialized transport proteins, such as MCT8, MCT10, and OATP1C1 (65). Intracellular trafficking involves intracellular binding substrates (IBSs) of thyroid hormones (e.g., CRYM) (66). These carriers appear to be necessary components of the feedback control, as simulated for IBS and demonstrated for MCT8 deficiency (27, 65). Additionally, in rodents their hypothalamic expression has been shown to be subject to regulation by T3 (67). Transmembrane transport control adds another layer of complexity to the system but is currently not well understood.

While negative thyroid hormone feedback mitigates thyroid hormone overproduction and hyperthyroidism, TRH is a potent defensive mechanism against undersupply, stimulating both pituitary TSH secretion and modulating its bioactivity (68–72). TSH stimulation of thyroid hormone production, in turn, is essential, because the TSH-independent basal capacity of the thyroid gland is limited and unable to maintain a euthyroid state. Tissue-specific glycosylation differentially regulates the hormone allowing for targeted signaling (73). Pars tuberalis-derived TSH has been shown to differ in its glycosylation pattern from the pars distalis-derived hormone, lacks the ability to stimulate the thyroid TSH receptor, and regulates deiodinase type 2 activity related to seasonality and behavior independent of thyroid hormone production (73). Long feedback control of TRH release by thyroid hormones involves both hypophysiotropic TRH neurons and tancytes, responding to humoral and neuronal inputs that can adjust the set point. The latter mechanism may integrate energy metabolism and thyroid function (74–76). This may play an important role in the pathogenesis of non-thyroidal illness (NTI) syndrome or thyroid allostasis in critical illness, tumors, uremia, and starvation (TACITUS) (74, 77–80).

Additionally, an ultrashort feedback loop involving the suppression of TSH by its own concentration has been proposed, mainly by one group (81–83). Our own studies including mathematical modeling of thyroid hormone homeostasis confirmed that this particular loop appears to be a relevant factor in influencing the TSH–FT4 relationship (8, 10).

The primary regulatory role of pituitary thyroid hormone feedback versus TRH stimulation has been studied using transgenic mice, but its relevance is still controversial (69, 70, 84, 85). In the pit-*D2* KO mouse, Fonseca et al. (86) demonstrated coordination between the hypothalamic and pituitary T3 pathways that involve type 2 deiodinase. The role of deiodinase in tancytes was increased in the absence of pituitary deiodinase in order to preserve euthyroid serum T3 levels (86). The selective loss of pituitary type deiodinase, while increasing basal TSH in the mouse, diminished TSH response to hypothyroidism (87). However, knock-out animals with various degrees of deficiencies in all types of deiodinase have suffered little as a consequence, being able to maintain sufficient homeostatic regulation (88–90). It appears that multiple adaptive layers exist to protect the basic functionality of the homeostatic feedback control from various challenges. Furthermore, a multitude of physiological and pathophysiological influences modulates the relationship between TSH and thyroid hormones at various sites of action, thereby influencing the location of the set point in health and disease (Table 2) (8, 9, 76, 91–111).

**Table 2:** **Physiological and pathophysiological influences that may modulate the relationship between TSH and thyroid hormones**.

Factor	Main site of action	Predominant mechanism	Main effect	Reference
Age	Pituitary and hypothalamic	Altered sensitivity of thyroid hormone feedback	Diminished TSH response with increasing age	(9, 91–95)
BMI	Pituitary, hypothalamic, and adipose tissue	Central modulators (e.g., leptin) and hyperdeiodination	Hyperthyrotropinemia	(9, 96–99)
Time of day	Pituitary and deiodinases	Circadian TRH rhythm and ultrashort TSH feedback	Circadian rhythms of TSH and FT3 and pulsatile TSH release	(40, 100, 112–115)
Pregnancy	Thyroid gland	TSH receptor stimulation by placental factors (hCG)	Stimulation of thyroid hormone secretion and TSH suppression	(101, 109, 110)
Non-thyroidal illness	Multiple	Set point alteration	Low-T3/T4 and inappropriate TSH response	(116, 117)
Genetic polymorphism	Pituitary	Set point variation	TSH variation	(118, 119)
Epigenetics	Pituitary	Long-term set point alteration	Resetting the system	(120)
Thyroid state	Pituitary and hypothalamic	Variable TSH response depending on distance from putative optimum	Exaggerated response or dampening effect	(6, 8)
TSH quantity	Pituitary	Ultrashort feedback loop	TSH suppression	(8, 82)
TSH quality	Pars tuberalis and pars distalis	Tissue-specific glycosylation of TSH	TSH bioactivity	(72, 73)
TSH agonists or antagonists (TSH-R Ab and hCG)	Thyroid gland	TSH receptor stimulation or blockade	Thyroid hormone stimulation/inhibition and TSH suppression/stimulation	(68, 101, 109, 111)
TRH	Pituitary	TSH production and TSH glycosylation	TSH stimulation and bioactivity	(69, 70, 72)
Neuromodulators (dopamine and somatostatin)	Pituitary	Set point modulation	TSH	(76)
Leptin	Central and hypothalamus	TRH stimulation	TSH increase	(74)
Cytokines (interleukin-6)	Pituitary	TSH inhibition	TSH decrease	(106)
Cortisol and glucocorticoids	Pituitary	TSH inhibition	TSH suppression	(104)
Deiodinase type 2	Central, hypothalamus, and pituitary	T4–T3 conversion	Sensitive feedback regulation by T4	(60, 75, 86)
Deiodinase type 1	Peripheral tissues	T4–T3 conversion	T3 generation	(57)
MCT8 and MCT10	Hypothalamus and pituitary	T3-dependent mRNA expression and thyroid hormone transport	Intra- versus extracellular thyroid hormone gradient	(65, 67)
CRYM	All cells	Intracellular binding substrate (IBS)	Intracellular thyroid hormone trafficking	(66)
Thyroid hormone receptor (TR) β2	Pituitary and hypothylamus	T3 binding	Receptor occupancy	(48)
TR costimulator cosuppressor (RXR)	Pituitary and hypothylamus	T3 binding	Receptor occupancy	(54, 71)
Iodine supply and iodine deficiency	Thyroid gland and autonomously functioning thyroid nodule(s)	Thyroid volume-related TSH response and TSH receptor or G protein mutations	TSH increase/decrease	(107, 111)
l-T4 treatment	Pituitary	Altered thyroid hormone feedback and set point	TSH–FT3 disjoint and FT3–FT4 dissociation	(121, 122)
Other thyroid-related compounds or drugs	Multiple sites	Thyroid inhibitors, thyroid mimetica, and endocrine disruptors	Changes in TSH, FT3, and FT4 and inhibition of conversion or T3 actions	(102, 103)

Complementing the idea of a multifaceted feedback control, we have recently proposed that a feedforward motif may also be operative, directly linking TSH with deiodinase activity and the control of corporeal conversion from T4 to T3 (123). While this study provides the first documentation for a TSH-deiodinase inter-relation in humans *in vivo*, the responsiveness of deiodinase type 1 and type 2 to TSH, presumably through a TSH receptor- and cAMP-dependent mechanism, has been well recognized (55, 124–130).

**Figure 3:**
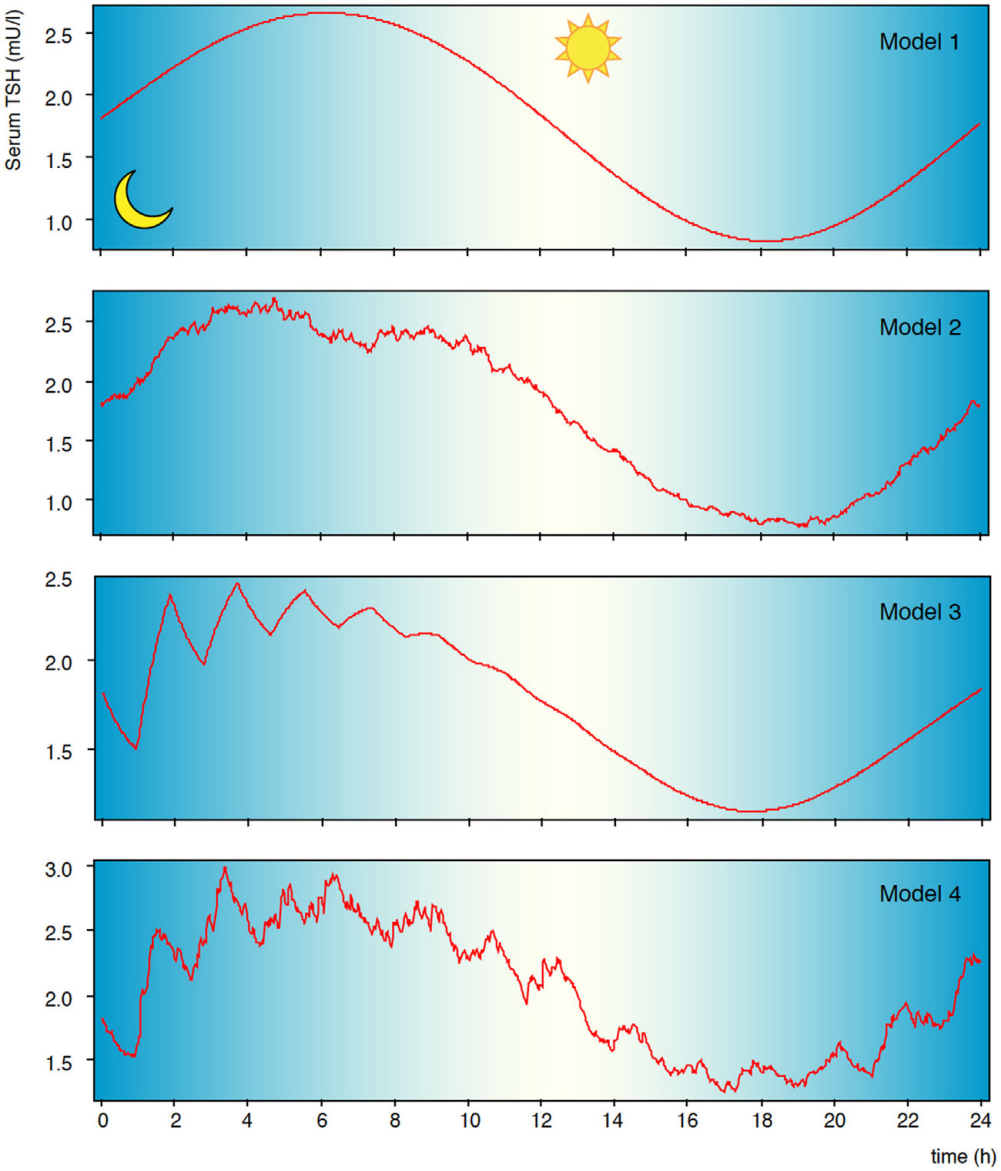
**Pulsatility of TSH secretion**. Secretion of thyrotropin is subject to circadian and ultradian variation. Shown are results of computer simulations with circadian input only (model 1), additional stochastic afferences (model 2), additional ultrashort feedback of TSH secretion (model 3), and combined stochastic input and ultrashort feedback (model 4). Statistical properties and fractal geometry of model 4 is identical to that of natural time series, while the simpler models differ (10).

Like other glycoprotein hormones, TSH is secreted in a pulsatile manner. Faster oscillations with a mean pulse amplitude of 0.6 mIU/l and a rate of 5–20/24 h are superimposed on a circadian rhythm with maximum TSH levels shortly after midnight (Figure 3) (112, 113, 131). It is still debated whether fast TSH pulses emerge by pulsatile TRH input, which has been contradicted by Samuels et al. (114), through stochastic signals or via autocrine function of thyrotrophs, i.e. controlled oscillations emanating from ultrashort feedback (10, 40, 115). TSH pulsatility may be beneficial by preventing homologous desensitization of the thyrotropin receptor (132–134). This could partly explain why sialylated TSH has both prolonged half-life and reduced bioactivity (135, 136).

A direct TSH-deiodinase link may, at least partly, explain the T3 circadian rhythm accompanying that of TSH, while FT4 shows no such related circadian or seasonal rhythm (137–139). This response may be modulated by regulating TSH receptor density, as shown in rats with severe thyroid dysfunction (140).

Importantly, these mechanisms align the task of defending plasma FT3 with the central control system (123). Supply of T3 to peripheral tissues is therefore no longer to be seen exclusively as a locally and autonomously regulated process, rather as a part of an overarching, integrated, and central-peripheral control system that governs thyroid hormone signaling in both homeostatic and allostatic regulatory modes (123). This is particularly relevant for the treatment situation with levothyroxine, as discussed below (121).

Figure 1 presents a synopsis of central, thyroidal, and peripheral influences and their homeostatic integration. Taken together, the molecular mechanisms defining multiple feedback loops on several levels of organization, different time scales, and varying conditions of their optimum operation may explain the disproportional non-logarithmic behavior of the TSH–FT4 relationship (Figure 2) (6–9, 33, 34). They support a multistep regulation and functionally hierarchical model that has been proposed by our group (8). While we have focused on describing the essential principles, additional physiological contributors, such as ethnicity, gender, age, body mass, iodine intake, selenium supply, T4 treatment, genetic deiodinase polymorphisms, and many others, may all elaborate the complexity of the system. Thus, further fine-tuning of the adaptive responses occurs at both the central and peripheral levels (Table 2) (9, 74, 76, 91, 92, 95, 97–99, 118, 119, 141–144).

## Emerging Role of Non-Classical Thyroid Hormones

**Figure 4:**
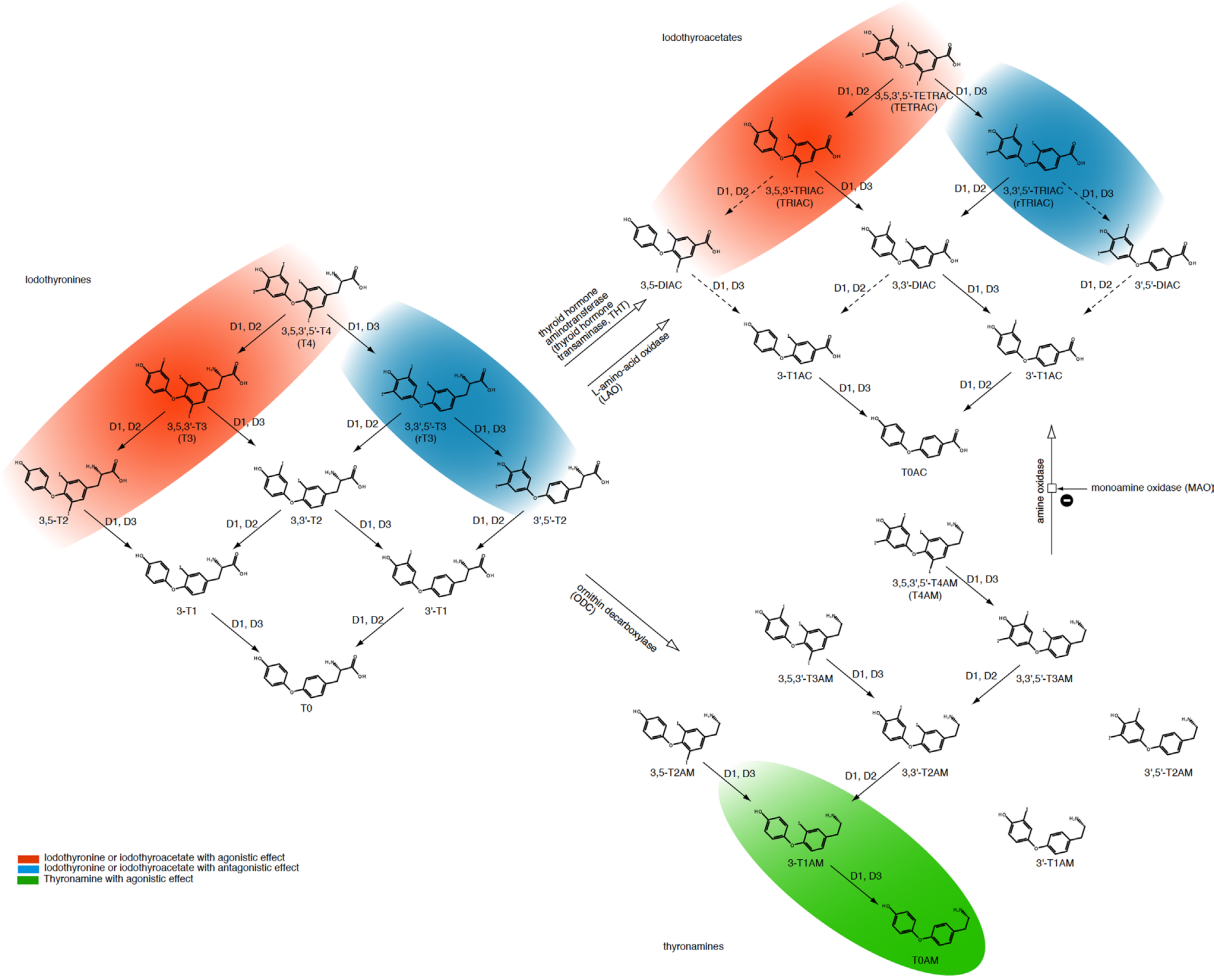
**Overview of classical (iodothyronines) and non-classical thyroid hormones (iodothyroacetates and thyronamines) with associated interconversion processes**. Adapted from Engler and Burger (152), Piehl et al. (153), Soffer et al. (154), and Hoefig et al. (155).

Some less recognized non-classical thyroid hormones, such as reverse triiodothyronine (rT3), 3,5-diiodothyronine (T2), iodothyroacetates, and thyronamines, have recently been revisited and found to play an active physiological role (Figure 4) (145, 146). rT3 (3,3′5′-T3) is a T3 isomer that is deiodinated in the 3′ position. It is upregulated in fetal life and NTI and interferes by blocking characteristics on thyroid signaling (147). 3,5-T2 exerts agonistic effects at nuclear thyroid hormone receptors, although its concentrations parallel those of rT3 in critical illness (148–150). Elevated 3,5-T2 concentrations in the non-thyroid illness syndrome could, at least in part, explain why patients displaying the low-T3 syndrome may not benefit from substitution therapy with l-thyroxine (l-T4) or l-triiodoythyronine (l-T3) (151).

Iodothyroacetates are smaller, deaminated variants of thyroid hormones and have similar effects to those of iodothyronines (152, 156). However, their plasma half lives and affinity to receptors and transporters differ from the latter (157, 158). Due to its smaller molecule size triiodothyroacetate (TRIAC) is used for the treatment of resistance to thyroid hormone (RTH), but this effect does not seem to be beneficial for all mutant variants (157, 158).

Although thyronamines originate from follicular thyroid tissue and are structurally similar to iodothyronines, their biological effects are different. In many respects, their effects are antagonistic effects to those of the classical thyroid hormones (153, 159, 160). Classical and non-classical thyroid hormones can be interconverted by enzymes in certain body compartments (Figure 4) (153–155). While the effect of non-classical thyroid hormones on the overall behavior of thyroid homeostasis is still to be elucidated in more detail, some molecules including 3,5-T2, TRIAC, and TETRAC have thyromimetic effects at TR-β receptors, thereby exerting TSH-suppressive actions (156, 157, 161–163). This suggests a role of non-classical thyroid hormones as important modulators of the overall control system in supporting feedback loops controlling release and conversion of thyrotropin and the classical thyroid hormones. The resulting complexity of the homeostatic system is reflected in the non-proportional relationship between FT4 and TSH concentrations (Figure 2).

## Consequences for Thyroid Function Testing

Accordingly, the novel insights into thyroid–pituitary hypothalamic regulation of thyroid hormones described above have important consequences for thyroid function testing. The initial discovery that pituitary TSH responds inversely to the underlying thyroid hormone concentration has greatly influenced current clinically applied thyroid testing (4). Its exaggerated response allows much greater sensitivity to subtle changes in the thyroid hormone status. The first TSH-based thyroid test strategies emerged in the 1980s (164). Whilst the vast majority of studies concentrated on TSH testing, there were few attempts at physiologically based modeling (Table 1) (10, 26, 101, 165, 166). The consensus of TSH as a more sensitive diagnostic test than FT4 measurement has been summarized repeatedly in laboratory-focused procedures on TSH measurement and clinical guidelines on its practical use (2, 4, 167). Technically, routinely employed TSH assays are now in their third generation with each advance significantly enhancing functional assay sensitivity and the ability to clearly separate suppressed TSH levels observed in overt hyperthyroidism from levels at the lower reference limits seen in euthyroid subjects (168). Clinicians have embraced the availability of such a sensitive and cost-effective instrument. TSH is also employed in numerous prognostic studies which define it as a statistical marker of future outcomes (169–175).

This has important consequences as to how TSH has become viewed by the thyroid community as a simple and efficient diagnostic parameter. The ease of measurement was translated into simplicity of interpretation, ignoring the fact that TSH is both an indirect measure reflective of thyroid hormone homeostasis and a controlling element. Thereby, this concept obscured the intricate relationship of the TSH response with the underlying change in the hormonal milieu. By separating TSH from its physiological roots and primary role as a controlling element (Figure 1), not only did it become a statistical parameter in its own right, but also it thereby gained the role of the dominant thyroid function test. Consequently, definitions of hypothyroidism or hyperthyroidism were adjusted, introducing new laboratory-based and TSH-derived disease entities of subclinical hypothyroidism and hyperthyroidism, which are defined by an abnormal TSH level while FT3 and FT4 still dwell within their reference ranges (176–178). This was a major conceptual shift, as a disease had now become exclusively defined by measuring a single laboratory value, and, as a result, thyroid disease prevalence was thereby linked to the performance of a single test (179).

## Homeostasis and the Reference Range of TSH

While acknowledging strategic advantages of TSH measurement, such as ease of use, suitability for first-line screening, detection of subtle functional abnormalities, and association with various health outcomes including mortality, there are considerable risks of distorting its integrated physiological importance. The misconception is highlighted by the ongoing controversy surrounding the reference limits of TSH, particularly its upper limit defining subclinical hypothyroidism (167, 180, 181). Proposed amendments to the range, taking into account additional factors such as hidden autoimmunity, ethnicity, gender, and age, offer minor corrections but still fall short of a satisfactory solution. The issues may be more fundamental in nature (182). Even logarithmic transformation of TSH does not totally succeed in restoring a normal distribution. Some authors have attributed this failure to the presence of hidden pathologies, such as autoimmune disorders, others disagreeing with that conclusion (183, 184). We have adopted an alternative statistical approach to the conventional method of establishing the reference interval (45). This involves extrapolation from a normally distributed, robust middle part of the range to the respective boundaries and is suitable for verifying proposed reference ranges by third parties, such as laboratories and manufacturers, using their own retrospective sample of the target population.

However, this does not overcome the problems of diagnostic interpretation using TSH. Unlike many other laboratory parameters, TSH values are personalized measures exhibiting a high degree of individuality. The ratio of the interindividual to the intraindividual variation may serve as an estimate of “individuality,” being much higher for TSH than most laboratory parameters, for example, 2 in an earlier report and 2.9 in a recent study (45, 185). Accordingly, the same TSH value could be “normal” for one individual but pathological for another. This also holds for patients with subclinical dysfunction, in whom the relationship between FT4 and TSH shows elements both of normality and abnormality (101). Apart from the statistical requirement that a TSH value in the subclinical range must change by 30% to be confidently classified as change rather than variation or fluctuation, the true nature of TSH referencing is bivariate in relation to an appropriate individual TSH level when combined with a certain FT4 level (101, 186–189). Pulsatility of TSH release adds to the intraindividual variation in TSH levels, which is higher than that of circulating FT4 concentrations (45, 190). Circadian and ultradian rhythms of TSH levels reduce diagnostic accuracy unless reference intervals are adapted or blood sampling is restricted to morning time (100).

Furthermore, the observed rather flat TSH–FT4 relationship within the euthyroid range of the population makes a particular TSH measurement more ambiguous in its prediction of the underlying thyroid state than it does when related by a steeper gradient (Figure 2). This questions reliance solely on TSH measurements whenever precise estimates of thyroid function are warranted, but to consider all three thyroid parameters TSH, FT4, and FT3 and their inter-relationships. However, only a few published studies have followed this approach, establishing as a proof of concept truly multivariate reference ranges for thyroid parameters, instead of a combination of two statistically independent univariate reference intervals (191–194). A model that respects the relationship between TSH and thyroid hormones raises the concept of an individually and conditionally determined set point. This is the intersecting point on the overlaid characteristic curves for thyroidal T4 production and pituitary TSH secretion (35, 41, 101). It is important to appreciate that the homeostatic relationship of TSH and FT4 defines the reference range of TSH in a “kite-shaped” graphical configuration, as opposed to the rectangular area obtained by plotting the two univariate parameters (101). A mathematical algorithm has recently been proposed to reconstruct the set point in an individual independently of a population-based reference range (35, 195). The clinical potential of this novel approach awaits further trials. It might prove useful in assessing the appropriateness of a TSH value in a given patient, thus legitimizing a personalized TSH target for thyroid hormone replacement therapy.

While relatively stable in thyroid health, the set point is, however, not fixed but acts as an important physiological integrator and modulator for the homeostatic and allostatic regulation of thyroid hormones (Figure 1) (9, 116). This demands a careful diagnostic interpretation taking into account additional information about the clinical condition and various historical influences that may temporarily or permanently impact on the location of the set point at various hierarchical levels (Table 2). In extremis, the notion of a non-fixed TSH set point is typified in the NTI syndrome and other constellations of thyroid allostasis where TSH measurement fails as a diagnostic test for that reason (117). The persistence of a significant homeostatic deviation for a prolonged period of time, may, in turn, irrevocably alter the position of the set point, which then assumes a “normality” that is now vigorously defended anew (120). This potential plasticity of thyroid homeostasis is part of a broader concept of epigenetic influences where the bidirectional interchange between heredity and the environment plays a defining role.

In conclusion, the conventional reference system and reliability of TSH measurement as a clinically adequate measure of euthyroidism is compromised by its indirect influence dependent on its fundamental relationship with the underlying thyroid hormones. This, however, is neither proportional (log-linear), as previously thought, nor is it unconditional, but rather complex, hierarchical, and highly individual. Consequently, subclinically hypothyroid patients therefore comprise a heterogeneous population of truly dysfunctional and truly euthyroid subjects. Hence, current definitions of subclinical hypothyroidism or hyperthyroidism cannot serve as a satisfactory and consistent aid to an accurate disease classification in itself. Emerging integrated and personalized diagnostic concepts need to be evaluated and appropriate new markers of tissue euthyroidism must be developed.

## Homeostatic Considerations in T4-Treated Patients

**Figure 5:**
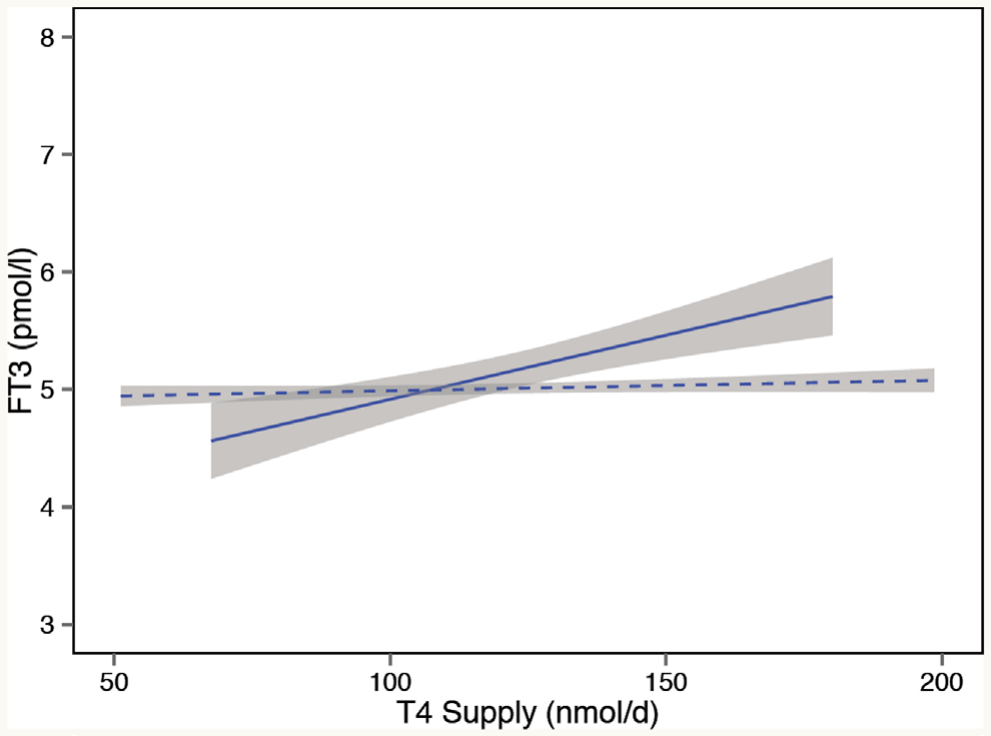
**Loss of T3 stability in l-T4-treated athyreotic patients with thyroid carcinomas**. In controls (dashed line), serum T3 remains stable over a wide variation in the endogenous thyroid hormone production. In contrast, in l-T4-treated patients (solid line), compensatory T3 regulation is broken, and serum T3 unstably varies with the exogenous T4 supply. Adapted and reproduced with permission from Hoermann et al. (123).

The rationale for using TSH as an important treatment target is that the patient’s own pituitary gland is generally assumed to be a good determinant for establishing the dose adequacy of l-T4 treatment, even though differences between the T3 utilization of various tissues may exist (4, 5). Thereby, the TSH value derived from optimum health is deemed an appropriate level to aim at for treatment in most patients, excluding systemic NTI and pituitary disorders where the TSH response is compromised. However, when put to the test, we and others found this assumption to be invalid (9). On the contrary, the interlocking inter-relationships between FT3, FT4, and TSH were not invariably fixed but conditionally and homeostatically determined. In l-T4-treated patients, we showed a significant upward or downward shift and change in the gradients of the FT4 or FT3 regression line with logTSH, compared to untreated controls (9). The phenomenon reveals a disjoint in the relationship between TSH and FT3 (122). While earlier studies already hinted that TSH normalization may not suffice to guarantee a normal serum T3, the more detailed inter-relationships have only recently been analyzed (9, 122, 196–199). From a homeostatic point of view, evidence suggests that the stability of serum T3 is maintained over a wide variation in the endogenous thyroid hormone production in healthy subjects but is lost in the l-T4-treated athyreotic patient (Figure 5) (121, 123). This observation raises questions regarding T3 adequacy in treated hypothyroidism. The lower FT3 levels frequently documented in athyreotic l-T4-treated patients, as compared to untreated controls, have received scant attention, being widely dismissed as easily compensated at the tissue level in humans. However, feedback regulation seems clearly compromised in athyreotic patients on l-T4 treatment resulting in T3 instability and homeostatic equilibria that differ significantly from those in healthy subjects (123) (Figure 5). In this respect, the presence of the thyroid gland itself and the size of the remnant thyroid tissue after thyroid surgery have recently been shown to play an important role in stabilizing serum FT3, presumably through TSH stimulated intrathyroidal T3 conversion (123, 200). This may explain why athyreotic patients are particularly vulnerable, with approximately 15% living in a chronically low-T3 state below reference, even if they are able to normalize TSH (122, 198). Three remarkable phenomena have been observed in l-T4-treated patients, (1) a dissociation between FT3 and FT4, (2) a disjoint between TSH and FT3, and (3) an l-T4-related conversion inefficiency (121, 123). Hence, l-T4 dose escalation may not invariably remedy T3 deficiency but could actually hinder its attainment (121, 201). In addition to substrate inhibition and an inhibitory action of reverse T3 on the enzyme activity of deiodinase type 2, experimental studies in the rat elaborate on molecular details involving ubiquitination that may explain a lack of effect of increasing T4 dose in this condition (64). In the rodent, FT3 concentrations in the circulation remained low after escalation of the l-T4 dose (64). More importantly, irrespective of local variations by tissue or type of deiodinase involved, tissue hypothyroidism persisted in all organs examined including brain, liver, and skeletal muscle despite a normal TSH (64). The recent findings are in agreement with earlier studies in the rat (64, 202, 203). T3 supply is locally controlled by several mechanisms, such as active thyroid hormone uptake, tissue-specific expression and activity of two distinct types of deiodinases converting T3 from T4, and thyroid hormone inactivation by deiodination or degradation by sulfatation, deamination, or glucoronidation (57–66). The regulation varies by tissue as the brain predominantly expresses type 2 deiodinase, whereas type 1 deiodinase is abundant in other tissues of the body, and by thyroid state, as T3 excess upregulates type 1 deiodinase but downregulates type 2 deiodinase, which is upregulated in hypothyroidism (57–64). Type 3 deiodinase produces T3 in an inactive form, reverse T3. Although T3 utilization may be locally adjusted to meet the specific demands of each organ, tissue supply is not autonomously independent, but subject to the overarching central control, as discussed above. While corresponding data on tissue T3 in humans are widely lacking and the physiological proportions of T3 derived by conversion versus thyroidal secretion may differ in humans and rodents, the animal models indicate widespread tissue hypothyroidism of target organs in the presence of low serum T3 and normal TSH. This suggests that the disequilibria recognized between circulating FT3 concentrations and both FT4 and TSH in patients on l-T4 may remain intracellularly uncompensated and truly reflective of tissue deficiencies. However, the long-term consequences of the altered ratios are presently unknown. Interestingly, a strong TSH–FT3 relationship was a marker of familial longevity in a recent study confirming the prognostically important role of the equilibria measured in the circulation (204).

The novel implications of homeostatic regulation require further careful study and clinical follow-up. In humans, quality of life may be reduced in a substantial portion of hypothyroid patients taking levothyroxine, even though normal TSH levels suggest restoration of euthyroidism (205). Importantly, the interpretation of TSH values is not uniform among different pathophysiological conditions. A given TSH value in an athyreotic patient on l-T4 has a diagnostic implication entirely different from the same value in an untreated euthyroid subject. While concentrating on treatment-related aspects of thyroid homeostasis, we have not specifically addressed the treatment of hypothyroidism, which has been covered by several recent specialized articles (205–211). Though optimum treatment targets and modalities invite a fuller debate and further research, it is, however, increasingly clear that l-T4 treatment in its current form, which lacks approximately 10% naturally secreted T3 component, is at base an unphysiological treatment modality where the resulting homeostatic responses operate differently from normality. The diagnostic situation cannot therefore be judged by the same TSH-based criteria defining optimum health (9, 122).

Hence, we suggest that the use of TSH, valuable though it is in many situations, should be scaled back to a supporting role that is more appropriate to its conditional interplay with peripheral thyroid hormones. We emphasize that measurement and consideration of FT3 and conversion efficiency is equally important, particularly in known situations where TSH and FT3 dissociate. This reopens the debate on measurement of free thyroid hormones and encourages the identification of suitable biomarkers. While TSH assays are traceable to a single WHO standard, FT4 and especially FT3 methods are in urgent need of equivalent standardization and harmonization if they are to play a clinically acceptable role in an integrated concept (212). It remains general good clinical practice to only interpret laboratory tests in conjunction with a clinical assessment of the patient history and symptoms and to obtain appropriate confirmation or follow-up before commencing treatment. While TSH may be suitable for screening of asymptomatic conditions, the integrated interpretation of TSH, FT4, and FT3 and their conditional equilibria should benefit decision making, particular on dose adequacy of replacement therapy. Possible adverse effects of the homeostatic disequilibria that arise under the current standard treatment of l-T4 replacement also warrant careful study, and new treatment strategies should aim at maintaining more physiological equilibria.

## Summary and Future Outlook

The concept of thyroid homeostasis offers new perspectives to optimize the interpretation of thyroid function tests and minimize the diagnostic misuse of an isolated and inappropriate statistical interpretation of TSH. The latter approach has wrongly assumed a level of diagnostic certainty that is inherently lacking in this indirect, conditional, and highly individual measure of thyroid function. We have described a new integrative concept, in which TSH becomes a context-sensitive conditional variable but is neither a precise marker of euthyroidism nor optimal for the fine-tuning of thyroid control. TSH levels defined for optimum health may not apply in many l-T4-treated patients. Because of a discernible disjoint between FT3 and TSH concentrations in athyreotic patients, this can result in an inability of T4 monotherapy to adequately address their therapeutic needs. Unlike in the healthy subject with adequate correction, FT3 levels now become unstably dependent on exogenous T4 supply. Furthermore, the T4-related conversion inefficiency may outweigh the benefits of escalating the l-T4 dose in some patients. Homeostatic principles question the isolated interpretation and disease-defining diagnostic value of TSH measurements, hence promoting both a more personalized approach and consideration of diagnosis in a more conditional adaptive context.

These perspectives raise a variety of issues that warrant further exploration and require carefully designed clinical studies before advancing to broader clinical application. The questions relate to multivariate reference limits, personalized set point reconstructions, and the additional value of FT3 for defining thyroid status and assessing dose adequacy in thyroid hormone replacement. There also may be clinical consequences and long-term risks of an unphysiological FT3–FT4 ratio, FT3–TSH disjoint, and impaired deiodinase activity on l-T4 replacement, supporting a possible role of combined treatment with T3 and T4 in selected patients with poor conversion efficiency.

## Search Strategy

References for this review were identified through the authors’ personal files and searches of PubMed for articles published from January, 1971, to March, 2015, by the use of broader terms, such as thyroid homeostasis, feedback regulation, feedback control, reference interval, TSH, triiodothyronine, deiodinase, set point, and synonyms or combinations of the terms. Historically, relevant articles published between 1918 and 1971 were obtained through searches in the authors’ personal files, Google Scholar, and other Online Archives’ Collections. Articles published in English, French, and German were considered. Articles resulting from these searches and relevant references cited in those articles were reviewed. A more narrow search for homeostasis was done using the following terms: “pituitary AND thyroid AND (feedback OR homeostasis)” and “thyroid AND (simulation OR modeling).” Combined search strategy delivered more than 2000 articles to identify homeostatic models and mechanisms related to thyroid–pituitary feedback. To narrow down the listed references from the vast literature found, individual articles deemed highly original and most relevant were included, and broader concepts were covered by comprehensive review articles whenever possible.

## Author Contributions

The authors jointly collaborated in conception of this review article, literature research, interpreting and condensing the results, and drafting of the manuscript. RH, JM, and RL provided Figures 2 and 5, and JD and RH created Figures 1 and 4 and Table 1.

## Conflict of Interest Statement

Johannes W. Dietrich received funding and personal fees by Sanofi-Henning, Hexal AG, and Pfizer and is co-owner of the intellectual property rights for the patent “System and Method for Deriving Parameters for Homeostatic Feedback Control of an Individual” (Singapore Institute for Clinical Sciences, Biomedical Sciences Institutes, Application Number 201208940-5, WIPO number WO/2014/088516). All other authors declare that there is no conflict of interest that could be perceived as prejudicing the impartiality of the research reported.
